# Anti-PD-1 Therapy—A Potential Treatment for Myocardial Metastasis From Nasopharyngeal Carcinoma: A Case Report

**DOI:** 10.3389/fimmu.2021.688682

**Published:** 2021-11-05

**Authors:** Xiaowan Tang, Weijun Zhou, Danjiang Huang, Lili Chen, Guangwen Zhang

**Affiliations:** ^1^ Department of Hematology and Oncology, Wenzhou Medical affiliated Huangyan Hospital, The First People’s Hospital of Taizhou, Taizhou, China; ^2^ Department of Clinical Medicine, Second Clinical Medical College, Wenzhou Medical University, Wenzhou, China; ^3^ Division of Radiology, Wenzhou Medical affiliated Huangyan Hospital, The First People’s Hospital of Taizhou, Taizhou, China

**Keywords:** nasopharyngeal carcinoma, head and neck tumor, myocardial metastasis, immunotherapy, anti-PD-1 therapy

## Abstract

Myocardial metastasis of nasopharyngeal carcinoma (NPC) is rarely reported in the literature. Some autopsy studies found metastases in more than 10% of cases with malignant neoplasm. However, patients are often diagnosed during the postmortem because myocardial metastasis is often asymptomatic, and its Cardiac complications tend to be severe and fatal. Patients with Cardiac metastases are often treated with chemotherapy or surgical intervention, although the prognosis is poor. Immunotherapy with anti-programmed cell death receptor-1 or ligand-1 (PD-1 or PD-L1) inhibitors has recently been reported to be therapeutically significant in multiple cancers, including melanoma, nonsmall cell lung cancer, and NPC, but the treatment of myocardial metastasis of NPC has not been reported. This study described the case of a 50-year-old male patient who presented initially with NPC and received radiotherapy as first-line therapy. For 20 years, he had recurrent Cardiac metastasis of NPC. The pathological examination suggested tPD-L1 expression. Therefore, off-label sintilimab (200 mg every 21 days) was administered. After 10 cycles of treatment, myocardial metastasis shrank and the enlarged mediastinal lymph nodes disappeared. This case report demonstrated that Cardiac metastasis of NPC expressing PD-L1 might have a sustained response to PD-L1 inhibitor–directed therapy.

## Introduction

Since 1917, Cardiac metastasis from malignant neoplasm has been reported, and some autopsy studies have shown more than 7.1% of patients with cancer presenting with Cardiac metastases (1, 2). The incidence among autopsies of head and neck (oral cavity, nasopharynx, pharynx, tonsil, larynx, and salivary gland) was 3.6% (3). However, patients are usually diagnosed during postmortem with an asymptomatic Cardiac invasion (4). Although the myocardial metastasis of nasopharyngeal carcinoma (NPC) is rarely reported, its incidence rate is the highest in South-East Asia, especially in some provinces of South-East China. In these regions, NPC is the sixth most common cancer in male patients, particularly among the Chinese and Malay populations (5). NPC is characterized by a high frequency of nodal metastasis. The most common distal metastases of NPC are the bones, lungs, and liver; cases with distal metastases to the heart are extremely rare, with only three reported cases (6–8). Consistent with the epidemiological characteristics of NPC, all patients were Chinese.

Regarding NPC management, radiation therapy is the principal treatment for early-stage disease, and concurrent chemoradiation is the preferred modality in more advanced cases. Platinum-based chemotherapy is the first-line treatment in patients with metastatic disease. Recently, immunotherapy has become a promising therapeutic approach for NPC, including adoptive T-cell therapy, Epstein–Barr virus (EBV)-directed vaccination, and immune checkpoint blockades (9). Immune checkpoint inhibitors have achieved breakthroughs in malignant neoplasm. NPCs are characterized by EBV infection (10), high programmed cell death ligand-1 (PD-L1) expression, and abundant infiltration of nonmalignant lymphocytes (11, 12). They can be potentially suitable for immune checkpoint treatment. Several clinical trials evaluating anti-programmed cell death receptor-1 (PD-1) monoclonal antibodies in recurrent or metastatic NPC have shown a promising clinical curative effect in immune checkpoint treatment (13–15). This study described the case of a 50-year-old man with myocardial metastasis from NPC, who achieved an ongoing major partial response with the PD-1 inhibitor sintilimab.

## Case Presentation

**Figure 1:**
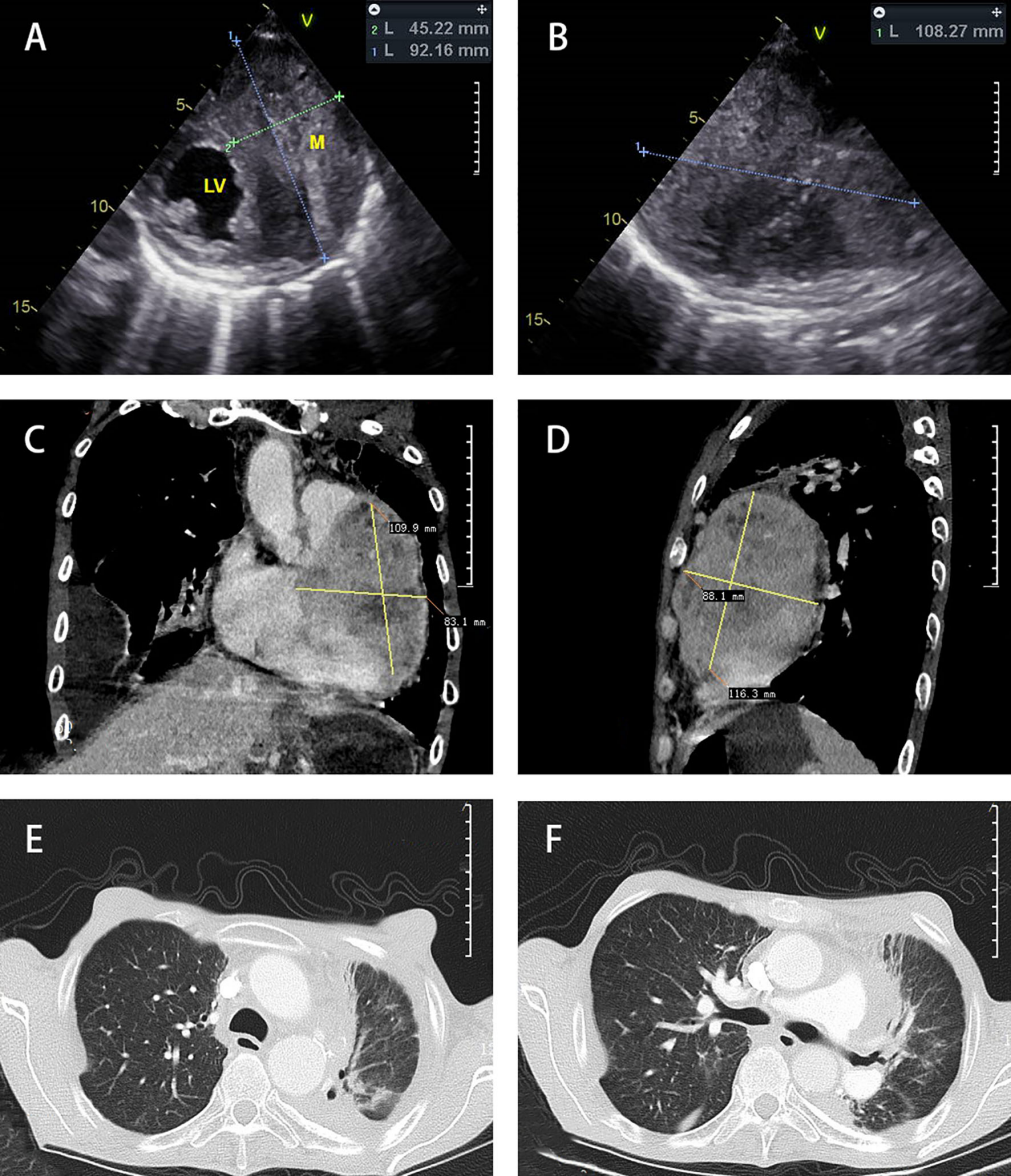
Diagnostic imaging. Echocardiography **(A, B)** showed a hypoechoic mass in the left ventricle and the anterolateral right ventricle. Chest CT scan mediastinal window **(C, D)** with contrast enhancement showed that the heart shadow had increased. A round, soft-tissue mass was observed at the left edge of the Cardiac margin. Multiple lymph node shadows were observed at the left hilum of the lung and mediastinum. The chest CT scan lung window **(E, F)** showed right pleural effusion and bilateral pneumonia.

In February 2019, a 50-year-old Chinese male patient visited our department due to a gradual onset of shortness of breath and palpitations. He was initially diagnosed with nonkeratinizing NPC in 1999 and had complete resolution of symptoms after radiotherapy and systemic chemotherapy (concrete primary treatment and staging was unknown). Overall health parameters during treatments were recorded (**Table 1**). Echocardiography suggested a hypoechoic mass spanning the left ventricle and the anterolateral right ventricle (92 × 45 × 108 mm^3^). The boundary between the left ventricle wall and the muscular layer was unclear (the upper part reached the level of the main pulmonary artery, and the lower part reached the level of the apex of the heart), and the arterial blood flow signal was detected (**Figures 1A, B**). The chest computerized Tomography (CT) scan with contrast enhancement showed an increase in heart shadow. Furthermore, a round, soft-tissue mass of 88 × 83 × 109 mm^3^ appeared at the left edge of the Cardiac margin, with enhanced heterogeneity. Multiple lymph node shadows were observed at the left hilum of the lung and mediastinum, and the left coronary artery was wrapped (**Figures 1C, D**). According to the 8th International Union Against Cancer (UICC) TNM Classification of NPC, the complete patient staging was TxN1M1, IVB Stage. Besides, the CT scan suggested pneumonia in the upper left lung, multiple lung infections, and right pleural effusion (**Figures 1E, F**). Considering the rarity of the case, a multidisciplinary team discussion was conducted with Ultrasonic Department, Radiology Department, Cardiovascular Department, Otorhinolaryngologic Department, Department of Thoracic Surgery, as well as Oncology Department. We both arrived at the same conclusion: the Cardiac hypoechoic mass was most likely the metastasis of NPC, and a pathological examination was needed.

**Table 1:** Overall health parameters during treatments.

Time	Feb-2019	Mar-2019	Apr-2019	May-2019	Jun-2019	Oct-2019	Feb-2020	Mar-2020	Apr-2020	May-2020	Jun-2020	Jul-2020	Aug-2020	Sep-2020	Oct-2020
Treatment	Admission	4 cycles of Sindirizumab treatment				Suspend	4 cycles of Sindirizumab treatment						Suspend	Pneumonia	
**Temp./℃**	37.4	36.6	36.2	36.5	36.4	36.9	35	36.9	36	36.2	36.2	36.6	36.9	36.2	36.7
**H.R./Min.**	130	120	120	112	106	109	96	103	95	95	99	99	95	110	114
**R.R./Min.**	32	27	22	22	20	20	20	20	20	20	20	20	20	24	27
**B.P./mmHg**	128/85	105/77	103/68	101/65	99/63	98/66	130/70	98/70	89/65	93/40	104/79	91/63	93/65	96/63	82/50
**Weight/kg**	46	45	45	45	45	45	45	49	49	45	42.5	45	45	43	42
**WBC/10^9^ L^-1^**	11.0	11.4	9.2	9.3	837	8.5	11.7	8.1	8.4	7.0	9.8	10.9	10.9	14.1	14.7
**HB/g L^-1^**	96	101	97	94	99	92	90	97	95	96	89	87	115	152	107
**BNP/pg mL^-1^**	529.7	NA	NA	NA	NA	NA	124	NA	NA	105	NA	NA	157	493	9790

**Figure 2:**
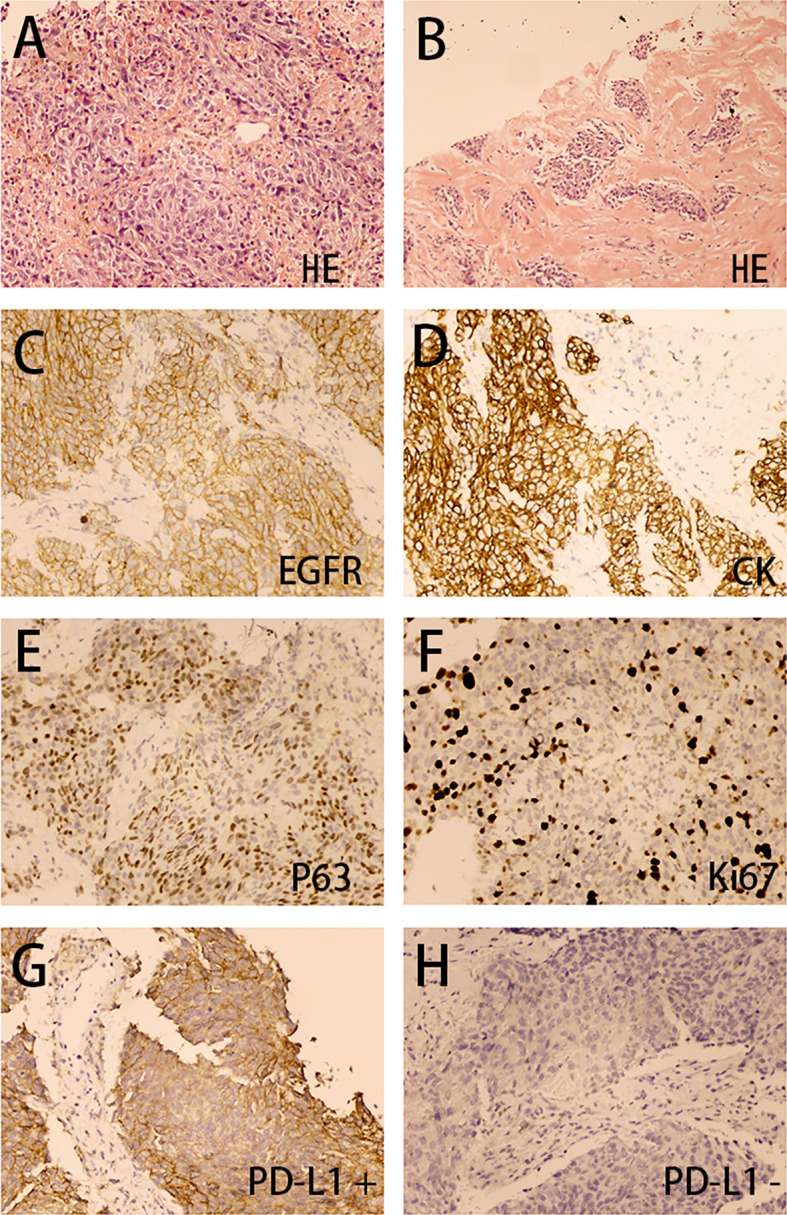
Right paraventricular mass biopsy (200×). The H&E stain **(A, B)** showed poorly differentiated carcinoma infiltration in fibrous tissue. The immunohistochemical analysis **(C–H)** showed EGFR (+), CK (+), P63 (+), Ki67 (40%+), PD-L1 (CPS 80%+), and PD-L1 (–).

A Cardiac biopsy was performed in March 2019 for further diagnosis. The right paraventricular mass biopsy showed poorly differentiated carcinoma infiltration in fibrous tissue (**Figures 2A, B**). Immunohistochemistry showed (EGFR) (+) (**Figure 2C**), CK (+) (**Figure 2D**), P63 (+) (**Figure 2E**), and Ki67 (+40%) (**Figure 2F**), suggesting nonkeratinizing NPC metastasis. The staining of the patient’s tumor tissue with Roche SP142 revealed PD-L1 expression by the combined positive score (CPS) 80% staining positive (**Figures 2G, H**). Based on the PD-L1 positive expression, the off-label treatment with sintilimab at 200 mg every 21 days (following its recommended dosage) was initiated on March 15, 2019. Antibiotics were not prescribed because the patient had no obvious symptoms of respiratory infection. After completion of four cycles, immunotherapy was terminated because the patient achieved major partial response and had limited paying capacity.

**Figure 3:**
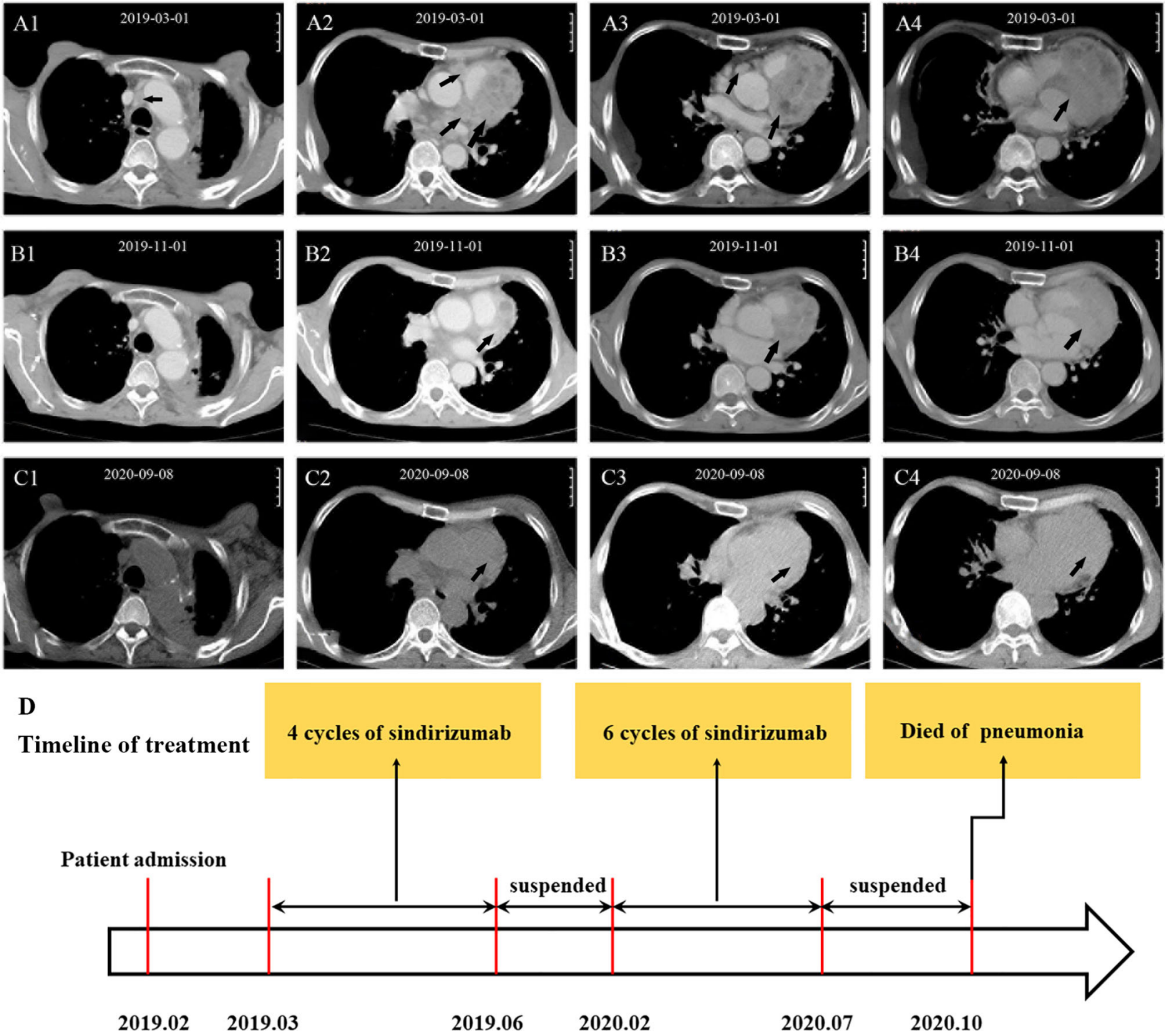
CT scans before and after treatment and timeline of treatment. Before treatment **(A1–A4)**: The arrows show multiple lymph node shadows at the left hilum of the lung and mediastinum and a round, soft-tissue mass at the left edge of the Cardiac margin. After treatment **(B1–B4)**: The arrows show that the enlarged mediastinal lymph nodes disappeared and myocardial metastasis significantly shrank. Follow-up **(C1–C4)**: myocardial metastasis continuously shrank. Timeline of treatment of the patient **(D)**: The arrow shows the whole process of diagnosis and treatment of the patient.

However, in February 2020, the patient received another six cycles of sindirizumab treatment again due to left axillary lymph node enlargement. Luckily, no obvious adverse events were observed during the whole therapy. The immunotherapy was continued till July 7, 2020, when the patient refused to undergo a contrast-enhanced CT scan on account of family conditions and personal worries about the side effects of the contrast agents. On September 8, 2020, the chest CT scan was performed, which revealed a marked shrinkage of myocardial metastasis and the disappearance of enlarged mediastinal lymph nodes, compared with the results on November 1, 2019 (**Figure 3**). However, due to constraints caused by hospital equipment and patient’s unwillingness, no more Cardiac biopsy was conducted, including immune infiltrate and changes during treatment. From May 2019 to September 2020, the patient had an Eastern Cooperative Oncology Group (ECOG) score of 0 and was allowed to engage in daily physical activity. No shortness of breath and palpitations were recorded during 18 months of follow-up. On September 14, 2020, the patient was admitted to hospital with a lung infection; the sputum culture showed the growth of various drug-resistant bacteria, including *Pseudomonas aeruginosa*, *Acinetobacter baumannii*, and *Candida albicans*. After combined antibiotic treatment and active rescue, the patient died in the Intensive Care Unit (ICU) due to severe pneumonia on October 26, 2020 (**Figure 3D**). In view of the history of bilateral pneumonia according to the chest CT images before the immunotherapy (**Figure 1F**) and sputum culture results, our first consideration was that the patient’s death was related to severe bacterial pneumonia, instead of the adverse effects of immunotherapy.

## Discussion

### Literature Review of Nasopharyngeal Carcinoma

NPC is one of the most common head and neck cancers in north Africa and southeast Asia, especially southern China (16). The worldwide distribution of NPC in 2018 indicated that the estimated number of new cases in China was 60,558 (47.7%) (9). Unlike other head and neck cancers, endemic NPC is closely associated with EBV infection, characterized by dense infiltration of lymphocytes in tumor stroma and positive PD-L1 expression in tumor cells, making it an attractive target for immunotherapy, especially immune checkpoint inhibitors (17). NPC exhibits the highest propensity for distant metastasis. Bones (48%), distant lymph nodes (43%), liver (36%), and lungs (31%) were the most common sites of distant metastases. In contrast, Cardiac metastasis of NPC is rare and often associated with poor prognosis (8).

### Cardiac Metastasis of Nasopharyngeal Carcinoma: A Rare and Fatal Disease

Primary tumors of the heart and pericardium are extremely rare, whereas secondary or metastatic Cardiac tumors are not uncommon. Peter et al. presented an extensive myocardial involvement case in which the patient with Cardiac metastasis was treated with surgical intervention under cardiopulmonary bypass, radiotherapy to heart, and systemic chemotherapy (6). Li et al. reported a patient with pericardial involvement who died suddenly before the pericardiocentesis procedure could be performed (7). Chen et al. reported a patient with pericardial metastasis who died of progressive disease after four courses of palliative chemotherapy with fluorouracil and leucovorin (8). Interestingly, all patients in these reports were Chinese. A summary of these three cases revealed that the first patient was successfully treated, but the treatment involved complex medical intervention, incurring an enormous cost and great suffering to the patient; however, the treatment failed in the other two patients. Thus, more effective therapy for Cardiac metastasis of NPC is urgently needed.

### A Potential Therapy for Cardiac Metastasis of NPC: Anti-PD-1 Therapy

Upregulation of the programmed cell death receptor-1 and ligand (PD-1/PD-L1) pathway is one of many possible mechanisms of immune evasion relevant to many cancers. Anti-PD-1 therapy is known to greatly prolong overall survival (18). Additionally, the anti-PD-1 regimen has a better response rate and a lower incidence of adverse events compared with chemotherapy (19). In our report, the staining of metastatic tumor tissue from the patient with NPC and Cardiac metastasis showed that 80% of cells expressed PD-L1, and immunotherapy led to a remarkable curative effect. Sintilimab is a fully human IgG4 monoclonal anti-PD-1 antibody, which has been proven to be clinically beneficial against multiple solid tumors, including lung cancer, hepatocellular carcinoma, gastric cancer, cervical cancer, advanced intrahepatic cholangiocarcinoma, and head and neck squamous cell carcinoma (20). Sintilimab has a similar antitumor effect, a better safety profile, and obvious economic advantages compared with nivolumab and pembrolizumab, the two well-studied PD-1 inhibitors approved by the China National Medical Products Administration (NMPA) and the US Food and Drug Administration (21). Thus, we chose sintilimab for our patient, who was a relatively rare case of Cardiac metastases from NPC with high PD-L1 expression confirmed by Cardiac biopsy. Importantly, the patient received 10 courses of irregular treatment (suspended for 8 months) and achieved a good curative effect. A better outcome might have been achieved if the patient took the medicine regularly and continuously. Although the overall response of the tumor to anti-PD-1 therapy was positive, further related studies are required for the following reasons: (1) The sample size of related cases is limited, and the underlying mechanism of anti-PD-1 therapy in our case remains unknown. (2) Whether the treatment can provide therapeutic efficacy in most Cardiac metastases of patients with NPC remains unclear. (3) A study showed that PD-L1 expression is upregulated in cardiomyocytes during inflammatory response and tissue damage in Cardiac injury models. However, in our patient, the myocardium was affected by the metastatic tumor but did not show high PD-L1 expression. (4) Whether this therapy can achieve a significant curative effect in patients with low PD-L1 expression remains unknown. In this case, PDL-1 was highly expressed in metastatic tumor cells in the myocardium, while PDL-1 expression was negative in adjacent cardiomyocytes. This difference should be further investigated. Metastasis in the heart is very rare, but is always lethal. Our case report provided preliminary evidence that anti-PD-1 therapy might be an effective and promising treatment option for some patients with high PD-L1 expression.

## Data Availability Statement

The datasets presented in this study can be found in online repositories. The names of the repository/repositories and accession number(s) can be found in the article/supplementary material.

## Ethics Statement

The studies involving human participants were reviewed and approved by Ethics Committee of Taizhou First People’s Hospital. The patients/participants provided their written informed consent to participate in this study. Written informed consent was obtained from the individual(s) for the publication of any potentially identifiable images or data included in this article.

## Author Contributions

GZ and LC contributed to the clinical care of the patient, designing the immunotherapy plan, and patient follow-up. XT and WZ contributed to the clinical care of the patient, editing of figures, data analysis, writing of the original draft, review and editing of the manuscript, and manuscript submission. DH contributed to radiography and editing of the manuscript. All authors contributed to the article and approved the submitted version.

## Conflict of Interest

The authors declare that the research was conducted in the absence of any commercial or financial relationships that could be construed as a potential conflict of interest.

## Publisher’s Note

All claims expressed in this article are solely those of the authors and do not necessarily represent those of their affiliated organizations, or those of the publisher, the editors and the reviewers. Any product that may be evaluated in this article, or claim that may be made by its manufacturer, is not guaranteed or endorsed by the publisher.
